# Lay theories of grandiose and vulnerable narcissism

**DOI:** 10.1007/s12144-020-01296-w

**Published:** 2021-01-19

**Authors:** Tatjana Koepernik, Emanuel Jauk, Philipp Kanske

**Affiliations:** 1 Clinical Psychology and Behavioral Neuroscience, Faculty of Psychology, Technische Universität Dresden, Dresden, Germany; 2 Department of Psychology, University of Graz, Graz, Austria; 3 Max Planck Institute for Human Cognitive and Brain Sciences, Leipzig, Germany

**Keywords:** Grandiose narcissism, Vulnerable narcissism, Lay theories, Implicit theories, Parental overvaluation, Parental coldness

## Abstract

In public discourse, narcissism is often portrayed one-sidedly and overly negative, rendering a picture of narcissistic individuals as “toxic people” or “evil characters”. Beyond these salient associations, psychological theories point to a more complex phenomenon, and different developmental mechanisms are being discussed in relation to it. We investigated the prevalence of different implicit theories on narcissism including beliefs about its developmental antecedents. We put forward the question whether grandiose and vulnerable narcissistic behaviors are regarded as congruent or incongruent expressions of underlying feelings and motives, that is whether grandiose behavior is attributed to underlying grandiosity or underlying vulnerability, and vice versa. Results of an online survey (*N* = 177) show higher agreement with congruent rather than incongruent theories (i.e., grandiose narcissism is attributed to feelings of superiority rather than inferiority, vulnerable narcissism is attributed to inferiority rather than superiority). In line with this, participants displayed predominant beliefs in parental overvaluation as a developmental antecedent of grandiose narcissism/parental coldness as an antecedent of vulnerable narcissism. With higher self-reported prior knowledge of narcissism, endorsement of theories assuming incongruencies increased. The likability of narcissism was not associated with endorsement of the different implicit theories, but instead with perceivers’ own narcissism levels. Results suggest that laypeople employ an “it is what it seems”-heuristic facing both grandiose and vulnerable narcissistic behaviors and are less likely to attribute grandiose or vulnerable behavior to incongruent motivational states. Findings might help to better understand the public image of narcissism and its social consequences.

## Introduction

During the past decades, there has been a boost in psychological research on narcissism and public attention to it. Some have argued that narcissism increases in western societies and labeled it a “narcissism epidemic” (Twenge, Konrath, Foster, Campbell, & Bushman, 2008), while others have argued against that (Trzesniewski, Donnellan, & Robins, 2008; Wetzel et al., 2017). Whether or not narcissism itself increases, interest and awareness for the phenomenon have definitely grown. Researchers have thoroughly investigated and debated the structure, expression, antecedents, and individual as well as social consequences of narcissism (Pincus & Lukowitsky, 2010; Miller et al., 2016; Grapsas, Brummelman, Back, & Denissen, 2020). Despite the intense research, one aspect has received surprisingly little attention, namely how laypeople perceive narcissism, and how prevalent different implicit theories on narcissism are. The lay perception of narcissism seems of particular importance given that the phenomenon is frequently portrayed in a rather one-sided and negative fashion in public discourse, and implicit theories might shape the way we perceive and thus deal with narcissistic individuals (Levy, Chiu, & Hong, 2006; Haslam, 2017; Furnham, 1988). Here, we address this question by investigating lay perceptions of grandiose and vulnerable narcissistic behaviors, their developmental antecedents, and the likability of grandiose and vulnerable narcissistic traits. Finally, we also relate those to perceivers’ own narcissism levels.

## Lay Theories

In order to orient within our social environment, we implicitly develop or adapt theories that provide “common-sense” explanations for complex and/or ambiguous behaviors, commonly referred to as implicit theories or lay theories (Plaks, Levy, & Dweck, 2009; Furnham, 1988; Levy et al., 2006). In this context, implicit means that the explanatory models (“theories”) need not necessarily be consciously represented, and are derived from everyday experience rather than systematic observation. Implicit or lay theories are thus not commonly formalized, and do not necessarily refer to latent constructs such as narcissism, but rather operate at the levels of observable behavior: “lay theories are […] commonly called implicit theories […] due to the recognition that these beliefs often operate at an automatic rather than conscious level – people have assumptions, largely unexamined, about the world around them which guide their judgements, but which have rarely been articulated in careful detail or bolstered with rational argument” (Wilson & English, 2017, p. 17).

Theoretically, for every particular behavior, an infinite variety of interpretations can be found. In order to reduce that complexity and thereby feelings of uncertainty, lay theories – just like scientific theories – are simplifications or working models that help us understand and predict other peoples’ intentions and behavior, and adjust ours accordingly (Levy et al., 2006). As Levy and colleagues (2006) summarized, lay theories provide us with a feeling of control and predictability, but – unlike scientific theories – are unlikely to depict accurate and dependable representations of our social world. Since lay theories are usually of implicit rather than explicit nature, we are frequently unaware of the significant influence these theories can have on our perception, judgements, and social behavior (Furnham, 1988; Levy et al., 2006). Thus, lay theories – or implicit theories-on narcissism may have a significant influence on judgements and behaviors towards narcissistic people as well.

### Expression of Narcissism

According to social-cognitive models of narcissism, the overarching goal of narcissistic functioning is to maintain an inflated self by means of characteristic intra- and interpersonal self-regulatory strategies (McWilliams & Lependorf, 1990; Morf & Rhodewalt, 2001; Back, 2018). The core of different forms of narcissism can be characterized by entitlement and self-importance (Krizan & Herlache, 2017) in terms of a preoccupation with own interests and concerns as well as the feeling of being entitled to special privileges (Ackerman, Hands, Donnellan, Hopwood, & Witt, 2017). Beyond this common core, grandiose and vulnerable narcissism can be discerned as two dimensions or phenotypes, which is evident across a wide range of psychological literature (Cain, Pincus, & Ansell, 2008; Pincus & Lukowitsky, 2010; Wink, 1991). While grandiose narcissism is characterized by social dominance, excessive self-confidence, and subjective well-being, vulnerable narcissism is defined by withdrawal, shame, and hypersensitivity to rejection or criticism (Czarna, Zajenkowski, & Dufner, 2018; Miller et al., 2016; Russ, Shedler, Bradley, & Westen, 2008; Pincus & Lukowitsky, 2010; Ronningstam, 2005; Krizan & Herlache, 2017).

The trifurcated model of narcissism (Miller et al., 2016; Weiss, Campbell, Lynam, & Miller, 2019) posits that variation in grandiose and vulnerable narcissism can be explained by a three-factor solution, which is based on the Five Factor Model of personality (FFM; McCrae & Costa, 2003). According to this model, grandiose and vulnerable narcissism are both associated with low agreeableness – or interpersonal antagonism – which constitutes the core of both phenotypes. Grandiose narcissism is additionally associated with extraversion (Miller et al., 2013; Kaufman, Weiss, Miller, & Campbell, 2018), while vulnerable narcissism is mainly associated with neuroticism (Kaufman et al., 2018; Miller et al., 2018b) and – depending on the particular inventory – introversion (Jauk, Weigle, Lehmann, Benedek, & Neubauer, 2017).

Another level of complexity can be added to the taxonomy of different aspects of narcissism by considering overt and covert expressions of grandiose and vulnerable narcissism (Pincus & Lukowitsky, 2010). Here, overt refers to experience and behavior shared with others, whereas covert refers to nonshared private experiences such as feelings, motives, and needs. According to Pincus and Lukowitsky (2010), “clinical experience with narcissistic patients indicates they virtually always exhibit both covert and overt grandiosity and covert and overt vulnerability” (p. 430). Clinical theory thus suggests that behavior might be congruent in some cases or states, but incongruent in others. Grandiose behavior, following this view, could be indicative of an underlying grandiose state (congruent), but could also mask underlying feelings of vulnerability, as overtly displayed vulnerability could mask underlying feelings of grandiosity (incongruent).

**Fig. 1:**
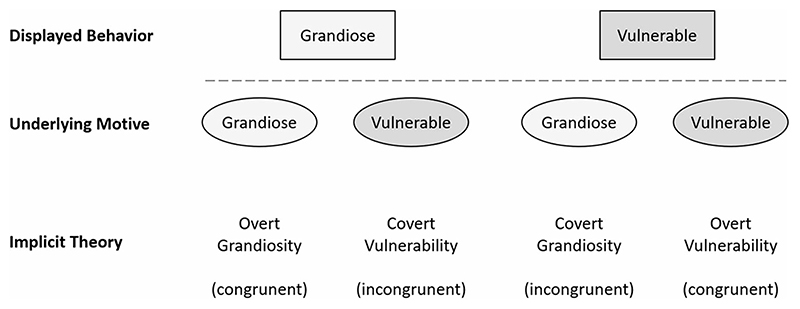
Congruent and incongruent implicit theories on grandiose and vulnerable narcissism

According to Miller, Lynam, Siedor, Crowe, and Campbell (2018a), laypeople perceive grandiose traits as most prototypic of narcissism. Beyond that, however, belief in narcissistic insecurity (BNI; i.e., attributing grandiose behavior to underlying vulnerability) is also prominent among laypeople (Stanton, Watson, & Clark, 2017). Here, we ask the question how prevalent congruent and incongruent lay theories on both grandiose and vulnerable narcissism are. To address this question, we systematically varied overt and covert expressions of grandiose and vulnerable narcissism in descriptions of narcissistic behavior. This allowed us to assess views about overt grandiosity and covert vulnerability as implicit theories on grandiose narcissism and overt vulnerability and covert grandiosity as implicit theories on vulnerable narcissism (see Fig. 1). As the detection of covert feelings and motives is commonly considered to require appropriate training (cf. Pincus and Lukowitsky, 2010), we hypothesize that in laypeople, implicit theories assuming congruency between displayed behavior and underlying motives (e.g., grandiose behavior as an expression of a grandiose state) will be more prevalent than implicit theories assuming incongruency (for instance: grandiose behavior as an expression of vulnerability).

### Developmental Antecedents of Narcissism

To investigate developmental aspects within implicit theories on grandiose and vulnerable narcissism, we assessed endorsement of the two most popular social-developmental theories of narcissism. These conceive parenting styles as particularly significant for the development of narcissism and can be briefly summarized as *parental overvaluation* (Millon, 1981) and *parental coldness* (Kernberg, 1975; Kohut, 1977). Parental overvaluation describes a parenting style that is characterized by excessive unwarranted praise and admiration. According to Millon (1981), children that are exposed to such noncontingent praise do not acquire the motivation and skills to work for these rewards in future situations, but rather develop a narcissistically entitled attitude. Parental coldness, on the contrary, circumscribes a cold, indifferent, and rejecting parenting style. Here, the development of narcissism is seen as a compensatory reaction to feeling invalidated and unloved (Kernberg, 1975; Kohut, 1977). Empirical research shows that parental overvaluation is indeed systematically related to the development of grandiose narcissism (Brummelman et al., 2015), and parental coldness is related to vulnerable narcissism (Otway & Vignoles, 2006). One of these studies also points to cross-associations in the way that both parenting styles are associated with both form of narcissism (Otway & Vignoles, 2006).

Wright and Furnham (2014) found that laypeople indeed deem dysfunctional parenting, such as excessive praise, lack of approval, or physical/mental abuse, to cause narcissism. Here, we aim to extend these findings by differentiating between grandiose and vulnerable narcissism. We hypothesize that laypeople associate grandiose narcissism primarily with parental overvaluation and vulnerable narcissism with parental coldness, as these associations are arguably most intuitive.

### Lay Theories and Likability of Narcissistic Traits

Lay theories on personality impact the way we perceive and judge others (Furnham, 1988; Levy et al., 2006). Aiming to go beyond cognitive models of others’ experience and behavior, we were also interested in the affective qualities associated with lay theories of narcissism. To our knowledge, these have to date not directly been investigated. We hypothesize that endorsing vulnerability as driving force of narcissistic behavior will be accompanied by a more positive evaluation than believing in underlying grandiosity. This could result from a more “sympathetic view of narcissism, as narcissistic behaviors may be considered to be more understandable if they are viewed as resulting from deep-rooted insecurity.” (Stanton, Watson, & Clark, 2017; p. 80). Similarly, endorsing parental coldness as a developmental antecedent of narcissism, rather than parental overvaluation, may lead to a more positive evaluation of narcissistic personality as well.

## Potentially Moderating Factors

### Knowledge of Narcissism

Prior knowledge of narcissism may influence the way we perceive narcissistic individuals. Wright and Furnham (2014) found that the prior knowledge about mental illness in general is positively associated with a belief in narcissistic fragility. To further understand whether fragility is attributed to grandiose or vulnerable aspects, however, a separate assessment of the two forms would be needed. Though research on prior knowledge of narcissism as a moderating factor in lay theories is limited, we tentatively hypothesize that individuals with higher prior knowledge will endorse implicit theories assuming incongruency between observable behavior and underlying motives (for instance: grandiose behavior as an expression of vulnerability) more frequently. We further investigate whether prior knowledge is also associated with likability of narcissistic personality.

### Rater Personality

The endorsement of different lay theories might not only be influenced by the degree of prior knowledge, but also by rater personality. There is evidence that individuals higher on grandiose narcissism show stronger belief in narcissistic insecurity (Stanton et al., 2017). Furthermore, Wright and Furnham’s (2014) findings suggest that raters’ narcissism influences their beliefs about causes of narcissistic personality. Specifically, individuals higher on narcissism endorsed early negative events as a cause of narcissism more frequently. We hypothesized that Stanton et al. (2017) as well as Wright and Furnham’s (2014) results would replicate here.

Moreover, we investigated effects of rater personality on likability of narcissism. Prior studies found that individuals higher on grandiose narcissism show higher tolerance for narcissistic traits in others (Adams, Hart, & Burton, 2015; Hart and Adams, 2014), which is why we expect higher likeability ratings of grandiose traits for individuals scoring higher on grandiose narcissism. We further examined whether endorsement of covert vulnerability underlying grandiose behavior plays a mediating role in the relationship between rater grandiose narcissism and likability of grandiose traits. To date, there is no research investigating the likeability of vulnerable narcissistic traits. We expected similar effects here.

## Hypotheses and Research Questions

Taken together, we hypothesized that (1) implicit theories assuming congruency between displayed behavior (including cognitions) and underlying motives are more prevalent than those assuming incongruency. Regarding developmental antecedents, (2) raters associate grandiose narcissism primarily with parental overvaluation, and vulnerable narcissism with parental coldness. We further assumed that (3) beliefs in underlying vulnerability (overt and covert vulnerability) as well as parental coldness lead to a more favorable evaluation of narcissistic personality traits than belies in underlying grandiosity or parental overvaluation. (4) We investigated the effects of prior knowledge of narcissism on lay theories and likability. We tentatively hypothesized that higher knowledge would be accompanied by relatively stronger endorsement of lay theories assuming incongruency between observable behavior and underlying motives. Further, we hypothesized that (5) raters’ own grandiose narcissism is accompanied by stronger endorsement of covert vulnerability and parental coldness, and (6) might also be associated with higher likeability ratings of others’ narcissism.

## Method

### Sample Size Estimation

To detect small to medium effects (r = .20, as common in personality psychology; cf. Gignac & Szodorai, 2016) in two-tailed tests at a power of 1-β = .80, a sample size of at least *N* = 191 individuals is required. We obtained an overall sample of *N* = 202 individuals, of which *n* = 25 had to be excluded (see below). Sensitivity analyses showed that with our final sample size of *N* = 177, effects equal to or bigger than *r* = .21 could be detected at a power of .80.

### Participants and Procedure

The final sample consisted of *N* = 177 German-speaking participants (59% female, 39% male, 2% not specified, *M_age_* = 36.43 (18-79 years), *SD_age_* = 16.08) who completed an online survey via LimeSurvey (www.limesurvey.org). This final sample was reached by excluding *n* = 25 participants who failed at least one of two attention checks (e.g. “Please check the box ‘very pleasant’”). The highest achieved educational levels were apprenticeship for 10%, secondary education for 6%, college degree for 35%, Bachelor’s degree for 15%, Master’s degree for 28%, and PhD for 6% of the sample.

Participants were recruited through a mailing list for study participation at Technische Universität Dresden, social media invitations, and Facebook posts in online survey groups. Before starting the survey, participants provided informed consent but were left blind to the study aims in order to prevent selection biases and socially desirable responding. Students of psychology were not approached since they would likely recognize the examined construct as narcissism, and might have theoretical knowledge about the construct, why their views cannot be considered lay perceptions. The study was approved by the university’s ethics committee.

The study duration was 26.9 (*Mdn)* minutes (*IQR* = 21.0–36.1 min). We assessed the study constructs in the following order: rater personality, lay theories on expression, lay theories on developmental antecedents, and likability. Items for the assessment of expression, developmental antecedents, and likeability of narcissism were randomized within each block. Self-report items for the assessment of rater personality were not randomized but presented in the order of the original questionnaires. After rating all items (see below), participants were asked about what they thought was assessed. Only 6% (*n* = 10) mentioned “narcissism” in their answers. We conclude that blinding was successful. Finally, participants were given information about the study objectives and asked about their prior knowledge of narcissism (from online sources [e.g. forums, videos], print media [newspapers, magazines], specialized books or textbooks, studies, professional education, or personal experience [self and social environment] and others’ shared experiences).

### Measures

Participants completed different item sets assessing their own narcissistic personality traits as well as lay theories and likability of narcissism. All were based on the Five-Factor Narcissism Inventory (FFNI; Glover, Miller, Lynam, Crego, & Widiger, 2012; Sherman et al., 2015), which provides the to date most comprehensive basis for the assessment of different aspects of narcissism grounded in the Five-Factor Model. For the evaluation of participants’ own traits, we administered the short form of the FFNI in its original form (see *Rater Personality*). For the assessment of lay theories and likeability, we adapted selected FFNI items for third-person ratings (see *Lay Theory and Likability Assessment* below).

#### Rater Personality

To determine raters’ degree of narcissism, we used the German 60-item short form of the Five Factor Narcissism Inventory (FFNI-SF; English original by Sherman et al., 2015; German translation provided by Mitja Back/ University of Münster). The FFNI-SF is a self-report measure consisting of 15 scales with a three-factor and a two-factor solution. The former comprises agentic extraversion, antagonism, and neuroticism while the latter reflects grandiose (11 subscales) and vulnerable narcissism (4 subscales). *Cronbach’s alpha* for the 15 subscales ranged from .70 to .91 (*Mdn* = .79), from .87 to .90 (*Mdn* = .90) for the three-factor-solution, and .88 and .92 (*Mdn* = .90) for the two-factor-solution. We additionally administered the German 21-item short version of the Big Five Inventory (BFI-K; Rammstedt & John, 2005), which is not analyzed here.

#### Lay Theory and Likability Assessment

The aim of our study was to investigate lay theories of expression and childhood antecedents of grandiose and vulnerable narcissism, and their effects on likability. We derived items that reflect opposing implicit theories of expression (overt grandiosity and covert vulnerability, overt vulnerability and covert grandiosity), developmental antecedents (parental overvaluation and parental coldness), and likability of grandiose and vulnerable narcissism from the well-established Five Factor Narcissism Inventory (FFNI; Glover et al., 2012; Sherman et al., 2015).

We selected 15 FFNI-items, one per subscale, from the FFNI-SF. Since the FFNI contains more grandiose than vulnerable subscales (11:4), we added another 3 items assessing vulnerable narcissism from the Pathological Narcissism Inventory (PNI; Pincus et al., 2009; German version by Morf et al., 2016). Items were chosen from the brief version (B-PNI; Schoenleber, Roche, Wetzel, Pincus, & Roberts, 2015), which contains the 28 best-performing items. We included the three scales *Contingent Self-esteem*, *Hiding the Self*, and *Devaluing*, which cover aspects of vulnerable narcissism that the FFNI does not include.

We selected items that had a simple structure (i.e., no negations, no conjunctions) and reformulated the item stems to the third person (e.g., “Someone who aspires for greatness” derived from “I aspire for greatness”). Next, we combined these item stems with different extensions, describing expression, developmental antecedents, and likability. Table 1 shows exemplary items for each item set, details on the construction of which are provided in the following.

##### Expression of Narcissism

Implicit theories on the expression and underlying motives of grandiose narcissism include overt grandiosity and covert vulnerability (see Fig. 1). Conversely, implicit theories on vulnerable narcissism include covert grandiosity and overt vulnerability. To assess these theories, we extended our item stems (derived from FFNI and PNI; see above) with statements either expressing beliefs in overt grandiosity/covert vulnerability (for grandiose narcissism), or overt vulnerability/covert grandiosity (for vulnerable narcissism). For instance, to assess overt grandiosity within the FFNI exhibitionism facet, we used the statement “when someone likes to be noticed by people, *it is because they consider themselves noteworthy*” whereas covert vulnerability for the same item was assessed with “*it is because they secretly feel too little regard*” in the second part of the statement (see Table 1). The 36 items were rated on a 5-point Likert scale (ranging from 1 ‘strongly disagree’ to 3 ‘undecided’ to 5 ‘strongly agree’).

##### Developmental Antecedents of Narcissism

To assess lay theories about developmental antecedents, we focused on the endorsement of either parental overvaluation or parental coldness as developmental roots of narcissism (cf. Otway & Vignoles, 2006). To obtain adequate descriptions of both parental coldness and overvaluation, we condensed the childhood recollection statements used by Otway and Vignoles (2006) into one clause each. Paralleling the item construction for the expression of narcissism, every item stem was then combined with both clauses. For example, the association of grandiose narcissism and parental over-valuation was assessed with the statement “when someone aspires for greatness, *this is a result of their parents idealizing them and putting them on a pedestal when they were a child.”.* To assess association with parental coldness, the same item stem was combined with “*this is a result of their parents being cold and treating them in an indifferent manner when they were a child.”* (see Table 1). The resulting 36 items were rated on the same 5-point scale (1 ‘strongly disagree’ to 3 ‘undecided’ to 5 ‘strongly agree’).

##### Likability of Narcissistic Personality

To assess likability of grandiose and vulnerable narcissistic behaviors, we used the third-person adaptations of the original FFNI items (complemented by PNI items; see above). Participants were asked to indicate how pleasant or unpleasant they would imagine an interaction with a person displaying the described attitudes or behaviors to be. The 18 items were rated on a 5-point Likert scale ranging from 1 ‘very unpleasant’ to 3 ‘neutral’ to 5 ‘very pleasant’.

### Analysis Plan

As preliminary analyses for the newly devised lay theory scales, we first determined item difficulties and internal consistencies, and then conducted confirmatory factor analyses (CFAs). We excluded items that were too easy or too hard and thus did not differentiate well between raters (i.e. *p* < .20 [raw mean < 1.8] or *p* > .80 [raw mean > 4.2]). We then compared the fit of different factor solutions using the *Root Mean Square Error of Approximation (RMSEA), Chi-Square Tests of Model Fit*, and the *Comparative Fit Index* (*CFI*). A good model fit is indicated by a non-significant χ^2^, CFI close to .95, and RMSEA <0.06 (Hu & Bentler, 1999).

Next, to test for mean differences in the endorsement of different lay theories on expression as well as developmental antecedents of narcissism (Hypotheses 1 and 2), we conducted two Bonferroni-corrected rm-ANOVAs. Analyses were conducted with 95% confidence intervals. To investigate the effects of prior knowledge (Hypothesis 4), we evaluated correlation patterns. We further planned to test three path models for mediational relationships between raters’ personality, lay theories, and likability (Hypotheses 3, 5, and 6). Since zeroorder correlations, however, showed little evidence for the hypothesized associations in the first place, these path analyses yield little new insight and are thus not presented.

## Results

### Confirmatory Factor Analyses of Lay Theory Assessment Scales

To test the assumed factor structure of the newly devised lay theory assessment scales, we conducted a series of CFA models, the results of which are displayed in Table 2. Grandiosity and vulnerability were modeled separately for each scale as the scales for developmental antecedents displayed too high correlations to include them into one model. For reasons of consistency, we kept this separation throughout all models. After inspecting a first series of models, we excluded the scale *Reactive Anger* (RA) from all vulnerability models, which improved model fit substantially for most models (except for *likeability of vulnerable personality*, where excluding *RA* lead to a slightly worse model fit. For reasons of consistency, however, we excluded the scale anyway^1^). For expression and developmental antecedents, we specified residual correlations between the two items with a shared item stem. Within the *likeability of grandiose narcissistic traits* scale, we excluded four items that were all rated very low on likeability and thus did not differentiate well between raters (*p* < .20 or raw *mean* < 1.8): *Arrogance (mean-* = 1.52), *Entitlement* (*mean* = 1.63), *Exploitativeness* (*mean* = 1.45), and *Manipulativeness* (*mean* = 1.75).

The final CFA models displayed mostly satisfactory fit to the data, though the χ^2^ test was significant for three scales, and the other indices were only partially within the desirable range for these same scales (see Table 2). Overall, however, the models yielded support for the theoretically assumed structure, why further analyses were conducted with average scores based on these CFA models.

### Descriptive Statistics

Table 3 shows means, standard deviations and correlations among raters’ personality, lay theories on expression, developmental antecedents, and likability of narcissistic personality. Means and correlations within the FFNI are generally similar to previous research (Jauk & Kaufman, 2018; Miller et al., 2016), indicating that the sample matches the general population on the variables of interest.

### Hypotheses Tests

To test for mean differences in the endorsement of different lay theories on expression as well as developmental antecedents of narcissism (Hypotheses 1 and 2), we conducted two rm-ANOVAs with Bonferroni correction, the results of which are shown in Table 4. Hypotheses 3 and 4, regarding associations between lay theories and likeability ratings as well as prior knowledge, were tested using correlations. Hypotheses 5 and 6 on the associations of lay theory endorsement and likeability ratings with rater personality were also tested using correlations.

#### Lay Theories on Expression of Narcissism

Our first hypothesis concerned the question whether grandiose and vulnerable narcissism are seen more as congruent rather than incongruent expressions of underlying feelings and motives (see Fig. 1). For this, we tested for mean differences within lay theories on expression of narcissism using a rm- ANOVA (see first two lines in Table 4).

We used Greenhouse-Geisser adjustment since Mauchly’s test indicated violation of sphericity (*χ^2^* (5) = 50.73, *p* < .001, Greenhouse-Geissers ε = .868). The omnibus test of mean differences was significant (F(2.60, 458.34) = 159.80, *p* < .001, Cohen’s *f* = .95), and pairwise comparisons showed significant differences between both pairs of variables. In line with our expectations, implicit theories assuming congruency between displayed behavior and underlying motives were endorsed significantly stronger than those assuming incongruency.

#### Lay Theories on Developmental Antecedents

The second rm-ANOVA tested for differences within perceived developmental antecedents of narcissism, namely whether grandiose narcissism is more frequently attributed to parental overvaluation than coldness, and whether vulnerable narcissism, on the other hand, is more frequently seen as a consequence of parental coldness rather than overvaluation. As Mauchly’s test again indicated violation of sphericity (χ^2^ (5) = 70.61, *p* < .001, Greenhouse-Geissers *ε* = .802), we used Greenhouse-Geisser adjustment here as well. The omnibus test was statistically significant (F(2.41, 423.44) = 170.24, *p*<.001, Cohen’s *f* = .98), and pairwise comparisons again showed significant differences in both variable pairs. In line with our hypotheses, laypeople’s endorsement of parental overvaluation as a causal factor of grandiosity was significantly higher than that of parental coldness, and the same was true for parental coldness as a factor causing vulnerability, as compared to parental overvaluation.

#### Associations Between Lay Theories and Likability

We hypothesized that beliefs in underlying vulnerability as well as parental coldness lead to a more favorable evaluation of narcissistic personality traits. To evaluate that, we inspected correlation patterns, as displayed in Table 3. Lay theories were generally unrelated to likability at conventional statistical thresholds. We observed small trends (*p* < .10) in the way that individuals who endorsed overt vulnerability as the source of vulnerable narcissism tended to view vulnerable narcissism as more likable, whereas individuals endorsing covert grandiosity as a source of vulnerable narcissism tended to find vulnerable narcissism more unlikable. However, as the effect sizes were small, the practical relevance of these findings might be limited.

#### Knowledge of Narcissism

To investigate whether prior knowledge of narcissism is related to the endorsement of the different implicit theories or likeability ratings, we inspected correlations among the respective measures (see Table 5). For the sake of clarity and comprehensibility, we built composite scores for correlated knowledge sources, in that we subsumed internet (e.g. forums, videos) and print media (newspapers, magazines) under *popular media*, textbooks, studies, and occupational knowledge under *academic/occupational knowledge*, and personal experience (self and social environment) and others’ stories under *personal experience*.

Table 5 shows that, in line with our expectations, greater knowledge in all three categories was significantly related to stronger endorsement of implicit theories assuming incongruency between observable behavior and underlying motives (grandiose behavior as an expression of vulnerability/vulnerable behavior as an expression of grandiosity). A similar yet weaker pattern of effects was evident for developmental antecedents in the way that individuals who reported more prior experience were more likely to endorse parental coldness as a causal factor of grandiosity and parental overvaluation as a causal factor of vulnerability. Likeability ratings were unrelated to prior knowledge.

#### Associations with Rater Personality

Further, we hypothesized that raters’ own grandiose narcissism is accompanied by stronger endorsement of covert vulnerability and parental coldness. To investigate this, we inspected the correlations between raters’ FFNI scores and lay theories. As Table 3 shows, endorsement of the different lay theories was generally unrelated to raters’ own narcissism as assessed by the FFNI. One unexpected exception to this was the correlation between raters’ grandiose narcissism and endorsement of covert grandiosity as a source of vulnerable behavior. When further evaluated within the three-factor model of narcissism, it can be seen that this effect related to the antagonistic (not extraverted) aspect of grandiose narcissism. Antagonism was further negatively associated with endorsing overt vulnerability as a source of vulnerable behavior.

Regarding likability of grandiose and vulnerable narcissism, we assumed that higher narcissism scores would be accompanied by higher likeability ratings of narcissistic traits. Correlations in Table 3 show that individuals higher on grandiosity/vulnerability also perceived these traits as more likeable in others. These effects were also reflected in the three-factor model of the FFNI in the way that those higher on agentic extraversion perceived grandiose traits as more likeable, whereas those higher on neuroticism perceived vulnerable traits as more likeable. Individuals higher on grandiose narcissism rated those higher on vulnerable narcissism as less likable.

### Discussion

This study set out to investigate lay theories of grandiose and vulnerable narcissism as well as the likability of narcissistic traits. In particular, we were interested in the extent to which laypeople endorse implicit theories assuming congruency or incongruency between observable behavior and underlying motives. We found that generally, implicit theories assuming congruency are more prevalent, but prevalence of theories assuming incongruency increases with prior knowledge. Implicit theories had little effect on the likability of narcissism, which was instead associated with raters’ own narcissism levels.

Regarding the public image of narcissism, our results support the idea that narcissism is frequently seen in a, so to speak, “one-sided” fashion. Grandiose behavior, for instance, is most likely to be seen as an expression of grandiose feelings and motives, whereas the view that vulnerable feelings and motives could also be at play (as discussed in psychological research; cf. Pincus & Lukowitsky, 2010) is less prevalent. However, the extent to which narcissism is seen as an expression of congruent or incongruent motives does not necessarily go along with more negative or positive views. We will elaborate on these findings in the following.

### Prevalence of Lay Theories on Expression and Developmental Antecedents of Narcissism

We hypothesized that implicit theories assuming congruency between displayed behavior and underlying motives are more prevalent than those assuming incongruency, which was confirmed in our study. This suggests that laypeople tend to adopt the most parsimonious explanation that still seems sufficient to explain others’ behavior, in terms of attributing observable behavior to congruent underlying emotional and motivational motives. Put simply, the prevailing heuristic for explaining narcissistic behavior seems to be “it is what it seems”. This heuristic might have adaptive value for many aspects of interpersonal behavior (e.g., it seems adaptive to assume that extraverted behavior indicates that someone desires contact), but this might not generalize to all aspects of human experience and behavior. Also, the finding that laypeople assume congruency between observable behavior and latent motives is in line with clinical perspectives on narcissism emphasizing the extensive training needed to sense potentially incongruent emotional and motivational motives underlying human behavior (cf. Pincus & Lukowitsky, 2010).

Concerning implicit theories on developmental antecedents, we observed a similar pattern of results, as the development of grandiose narcissism is mainly attributed to a parenting style characterized by overvaluation and unwarranted praise, whereas the development of vulnerable narcissism is attributed to coldness and emotional rejection. Again, lay theories seem to be dominated by intuitive and parsimonious models: if parents put their children on a pedestal, children might get spoiled and consider themselves worth more than others. If parents, conversely, behave indifferent and cold, a child might become insecure and distrustful. Again, these explanations appear to be very reasonable working models in many cases, as they also reflect the predominant effects which were found in systematic research (Brummelman et al., 2015; Otway & Vignoles, 2006). However, they might not consider the full complexity of developmental trajectories which are being scientifically discussed and studied, pointing to crossassociations of parental overvaluation with vulnerable narcissism and parental coldness with grandiose narcissism, though to a lesser extent (Otway & Vignoles, 2006).

### Do Lay Theories Impact the Likability of Narcissism?

We proposed that lay theories would be associated with the likability of narcissism. Particularly, we hypothesized that belief in vulnerability as a cause of underlying grandiose-narcissistic behavior would be accompanied by higher sympathy (or less antipathy) for individuals high on grandiose narcissism (cf. Stanton et al., 2017). This hypothesis was not supported by our data, as we did not observe a significant correlation between covert vulnerability as an implicit theory for grandiose-narcissistic behavior and ratings of likability of grandiose narcissism. Instead, we observed a-priori unexpected association of overt vulnerability and covert grandiosity as implicit theories for vulnerable narcissism and likeability of vulnerable narcissism. When people attributed vulnerable narcissism to overt vulnerability instead of covert grandiosity, by trend, they perceived vulnerable narcissism as more likeable. This could indicate that a vulnerable self is viewed as more likeable than a grandiose self. However, the effects were small, and we did not observe any similar pattern for grandiose narcissism. Also, lay theories on developmental antecedents were not associated with likability. To sum up, contrary to research emphasizing the significance of implicit theories for attitudes towards others (Furnham, 1988; Levy et al., 2006), the effects observed here for narcissism were small and inconsistent. Although this suggests little impact of implicit theories on imagined interpersonal relations in the first place, future studies could investigate whether lay theories modify actual interpersonal *behavior* towards narcissistic people. This might speak to how narcissistic individuals are treated rather than perceived.

### Knowledge of Narcissism

Higher self-reported prior knowledge of narcissism – no matter from what source (popular media, academic/occupational knowledge, or personal experience) – was accompanied by stronger endorsement of implicit theories assuming incongruence between observable behavior and underlying motives. Similarly, higher prior knowledge was associated with endorsing parental coldness as an antecedent of grandiose and parental overvaluation an antecedent of vulnerable narcissism. This demonstrates that prior knowledge adds complexity to lay theories on narcissism, in the way that less intuitive reasons and origins of narcissistic personality are considered.

Our findings differ from those of Wright and Furnham (2014), who found lay theories to be shaped by personality but – in most regards – not by the prior knowledge about mental illness. Contrarily, in our data, lay theories were associated with the level of prior knowledge. This discrepancy could be due to the fact that Wright and Furnham (2014) referred to the *general* knowledge about mental illness, while we assessed *specific* knowledge of and experience with narcissism. It is conceivable that specific knowledge of narcissism has a higher impact on lays’ narcissism theories than general mental illness literacy. Although, in our study, knowledgeability led to endorsing incongruencies, it did not influence how likeable narcissistic personality was perceived. However, as we assessed self-reports of prior knowledge, we can also not rule out the possibility that individuals who hold less intuitive views about narcissism simply rated their level of knowledge higher. Future studies could use a test of commonly accepted facts to evaluate this.

### Rater Personality and Lay Theories

Based on previous research demonstrating associations between grandiose narcissism and belief in narcissistic insecurity (Stanton et al., 2017) as well as experience of early negative events as a cause of narcissism (Wright & Furnham, 2014), we hypothesized that raters’ own grandiose narcissism would be accompanied by stronger endorsement of covert vulnerability and parental coldness as sources of grandiose narcissism. We found no evidence for these hypothesized relationships; our findings thus differ from those of Stanton et al. (2017) and Wright and Furnham (2014).

The discrepancy to previous findings might result from the overt (explicit) vs. covert (implicit) assessment of lay theories on narcissism: in both prior studies, narcissism was explicitly mentioned in some items. In our study, in contrast, we adapted items from a comprehensive narcissism inventory (FFNI) without explicitly naming the construct; the vast majority of participants did also not guess the study aim (see Methods). We think that our approach is well in line with the idea of *implicit* personality theories in an ecologically valid manner, as, in everyday life, we also observe others’ behavior, not psychological constructs. Future research could directly compare both assessment methods against each other.

Our data further indicated that raters’ grandiose narcissism is substantially related to endorsing covert grandiosity as a source of vulnerable behavior. This might either indicate that grandiose individuals are more sensitive in detecting covert grandiose motives in others, or it could also be indicative of projectively ascribing own motives to others. Future research could thus investigate the specificity of this finding by including non-narcissistic personality descriptions as well. Also, though the effect was among the strongest in our study, it was unexpected, and awaits replication in future studies.

### Rater Personality and Likability

While the likability of narcissistic traits was mostly independent of the raters’ beliefs about narcissism, the raters’ own narcissism was significantly associated with it. We found that those high on grandiose narcissism rated grandiose traits as more favorable, and those high on vulnerable narcissism rated vulnerable traits more favorable. Our study is thus in line with Adams et al. (2015) and Hart and Adams (2014) who found that individuals high on grandiose narcissism view grandiose personality traits more positively than those lower on narcissism. Beyond this, our research implicates that not only grandiose narcissists view their own personality traits more positively in others, but also vulnerable narcissists do so. Moreover, those high on grandiose narcissism viewed vulnerable narcissism more negatively. Taken together, similarity is associated with likeability in both cases. This might be the case because, on the one hand, it is easier to relate to similar others and understand their motives, and on the other, evaluating similar others favorably also puts the own personality into a more positive light.

### Limitations

Though our study provided encouraging insights on lay theories of grandiose and vulnerable narcissism, it is not without limitations. First, some of the scales that we adapted for third-person ratings did not display satisfactory model fit and internal consistency. While our approach to adapt an existing self-report inventory for the assessment of views about others’ personality ensures that different facets of the construct are adequately covered, for some of these facets, construction of novel items might be advantageous, and items could be tested for comprehensibility, refined, and validated.

Second, the sample studied here was not representative for the population, and it could be expected that sample characteristics might impact the endorsement of different lay theories as well as likeability ratings. To test for this possibility, we conducted complemental analyses controlling for participants’ sex, age, and prior diagnoses of mental disorders. Though the results were invariant with respect to these characteristics (see supplemental material S1), future studies could use larger and more representative samples to corroborate the present findings.

Third, as discussed above, we relied on a covert assessment of implicit theories without explicitly naming the narcissism construct. Though we believe that this approach has high ecological validity as it reflects real-life situations, it nonetheless differs from the assessments used in previous studies (Stanton et al., 2017; Wright & Furnham, 2014). Future research could thus directly contrast both assessment approaches.

Fourth, we used a self-report measure that provided participants with predefined statements as lay theories. While this allows for deductive tests of hypotheses, according to Nisbett and Wilson (1977), a shortcoming with self-report measures like this is that respondents might report more than they actually know, which could be avoided by using an open response format.

Lastly, though this is not a limitation of our study, we want to emphasize that the results obtained here cannot speak to whether a particular implicit theory is more accurate than another, or whether implicit theories assuming congruency or incongruency between displayed behavior and underlying motives are more adequate descriptions of narcissistic personality. While these questions need to be answered by personality and clinical research on narcissism, our findings can inform about the prevalence of different perspectives among laypeople. We think it is important for psychological research and also applied fields to know about laypeople’s views on narcissism, as these might reflect a public image which is seemingly more negative and emotionally charged than that of other clinically relevant personality configurations or mental disorders. While more research will be needed to systematically evaluate this assumption, we think our study can provide some insights which might be relevant to public discourse.

## Conclusion

The current study shed light on lay perceptions of grandiose and vulnerable narcissism, which is a largely uncharted field in psychological research. We found that lay theories attributing observable narcissistic behavior to congruent emotional and motivational motives have higher prevalence than those assuming incongruencies. Similarly, developmental antecedents of grandiose and vulnerable narcissism are predominantly seen in intuitive predictors: grandiose narcissism is mainly attributed to parental overvaluation while vulnerable narcissism is attributed to parental coldness. With increasing prior knowledge about narcissism, however, endorsement of implicit theories assuming incongruencies between observable behavior and underlying motives as well as less intuitive developmental antecedents also increased. Lastly, the likability of narcissism does not depend on lay theories but on raters’ own narcissism levels. Taken together, these findings support the idea that lay theories of narcissism rely on intuitive and parsimonious explanations, as feelings and motives which are incongruent to observable behavior are not commonly seen as driving forces of narcissistic behavior. However, this does not readily affect the likeability of narcissistic behaviors.

## Supplementary Material

ESM1

ESM2

ESM3

## Figures and Tables

**Table 1:** Exemplary items

Scale	Item stem	Extension
Expression		
Overt grandiosity (EXH)	When someone **likes to be noticed by people**	This is because they consider themselves noteworthy
Covert vulnerability (EXH)	When someone **likes to be noticed by people**	This is because they secretly feel too little regard
Overt vulnerability (DIS)	When someone **is slow to trust people**	This is because they are afraid to be disappointed
Covert grandiosity (DIS)	When someone **is slow to trust people**	This is because they have a low opinion of others
Developmental antecedents		
Parental overvaluation (AS)	When someone **aspires for greatness**	This results from their parents idealizing them and putting them on a pedestal when they were a child
Parental coldness (AS)	When someone **aspires for greatness**	This results from their parents being cold and treating them in an indifferent manner when they were a child
Parental overvaluation (SH)	When someone **feels ashamed when people judge them**	This results from their parents idealizing them and putting them on a pedestal when they were a child
Parental coldness (SH)	When someone **feels ashamed when people judge them**	This results from their parents being cold and treating them in an indifferent manner when they were a child
Likability		
Grandiose narcissism (MP)	Someone, who **is pretty good at manipulating people**,	Is […] to me
Vulnerable narcissism (NA)	Someone, who **often feels as if they need compliments from others in order to be sure of themself,**	Is […] to me

*Note.* These items are for a clearer visualization and were not used in our study that was conducted in German language. Item parts identical (except for conjugation) to the original items are printed in bold font. They were transformed from first to third person. *EXH* exhibitionism, *DIS* distrust, *AS* acclaim seeking, *SH* shame, *MP* manipulativeness, *NA* Need for Admiration

**Table 2:** Factor reliability and validity of self-constructed scales (N = 177)

Model	Factors	Indicators per factor	Correlation of factors (*p*)	Range of factor loadings	*RMSEA*		*Chi-Square*		*CFI*	*Cronbachs Alpha*
					*Est*.	95%-*CI*	*X^2^ (df)*	*P*		
Expression GN	OG	11	-.22 (.018)	.33-.64	.06	.05; .07	318.44 (197)	<.001	.83	.80
	CV	11		.19-.57						.73
Expression VN	OV	6	-.03 (.811)	.34-.50	.04	.00; .07	58.77 (47)	.117	.93	.55
	CG	6		.21-.64						.51
Antecedents GN	GN(PO)	11	.27 (.004)	.33-.63	.09	.08; .10	470.17 (197)	<.001	.77	.85
	GN(PC)	11		.30-.74						.76
Antecedents VN	VN(PO)	6	-.11 (.269)	.53-.73	.03	.00; .06	54.06 (47)	.223	.99	.64
	VN(PC)	6		.30-.66						.80
Likability GN	GN(L)	7		.13-.76	.04	.00; .09	17.36 (14)	.238	.97	.55
Likability VN	VN(L)	6		.17-.68	.09	.04; .14	21.77 (9)	.010	.90	.63

*Note*. Model fit according to Confirmatory Factor Analyses. *GN* grandiose narcissism, *VN* vulnerable narcissism, *OG* overt grandiosity, *CV* covert vulnerability, *OV* overt vulnerability, *CG* covert grandiosity, *PO* parental overvaluation, *PC* parental coldness, *L* likeability

**Table 3:** Means, standard deviations (SD) and Pearson correlation matrix of continuous variables (*N* = 177)

Variables	*Mean (SD)*	2	3	4	5	6	7	8	9	10	11	12	13	14	15
Self report measures																
Five factor narcissism inventory (FFNI)																
FFNI 2-factor solution																
(1) Grandiosity	2.25 (0.52)	**.18**	**.88**	**.83**	-.08	.03	.05	-.08	**.35**	.01	-.07	-.09	.11	**.33**	-**.21**
(2) Vulnerability	2.76 (0.70)		**.49**	**.22**	**.83**	.01	.06	.03	.02	.05	.06	.04	.09	-.05	**.19**
FFNI 3-factor solution																
(3) Antagonism	2.11 (0.53)			**.60**	**.15**	.03	.06	-**.15**	**.38**	.01	-.05	-.14	**.16**	**.23**	-.10
(4) Agentic extraversion	2.62 (0.68)				.11	.05	-.02	.02	.14	-.02	-.04	.01	.00	**.30**	-**.22**
(5) Neuroticism	3.11 (0.84)					.00	.04	.06	-**.18**	.02	-.02	.02	-.01	-.07	**.23**
Implicit theories/perception of narcissistic personality																
Grandiose expression
(6) Overt grandiosity	3.78 (0.55)						-**.16**	.14	**.28**	.03	**.33**	**.25**	.05	-.09	-.12
(7) Covert vulnerability Vulnerable expression	3.05 (0.56)							**.31**	**.17**	**.34**	.08	.00	**.41**	-.04	.01
(8) Overt vulnerability	3.61 (0.55)								-.09	.08	**.20**	**.23**	.04	-.12	.13
(9) Covert grandiosity	2.69 (0.59)									**.21**	.02	-.14	**.33**	.08	-.14
Developmental antecedents of grandiosity																
(10) Parental coldness	2.79 (0.59)										**.21**	**.39**	**.23**	.03	-.03
(11) Parental overvaluation	3.53 (0.67)											**.55**	.12	-.05	.04
Developmental antecedents of vulnerability
(12) Parental coldness	3.72 (0.73)												-.12	.02	-.01
(13) Parental overvaluation
Likability	2.55 (0.63)													-.07	-.01
(14) Grandiosity	2.59 (0.51)														-.03
(15) Vulnerability	2.85 (0.45)																

*Note.* Correlations above .25, .20, .15, and.13 are significant at *p* < .001, *p* <.01, p <.05, and p < .10 (two-tailed) respectively. Correlations significant at *p* < .05 are printed in bold type

**Table 4:** Pairwise comparisons of repeated measures ANOVAs (*N* = 177)

			95%-*CI*			*Significance (2-tailed)*	*Cohens’s d^b^)*
Expression/displayed behavior	Hypothesis	*Mean Diff*	*lower*	*upper*	*t*(176)		
Grandiose narcissism	OG-CV	0.73	0.57	0.90	11.58	<.001	1.32
	GN(PO)-GN(PC)	0.74	0.58	0.90	12.38	<.001	1.17
Vulnerable narcissism	OV-CG	0.92	0.75	1.10	14.53	<.001	1.62
	VN(PC)-VN(PO)	1.17	0.96	1.37	15.25	<.001	1.72

Formula: *ds* = (M2-*M1)/SD_pooled_*

b *Note.* Cohen’s *d_s_* with pooled standard deviation (adapted from Cohen, 1988)

*OG* overt grandiosity, *CV* covert vulnerability, *OV* overt vulnerability, *CG* covert grandiosity, *PO* parental overvaluation, *PC* parental coldness, *GN* grandiose narcissism, *VN* vulnerable narcissism

**Table 5:** Means, standard deviations (SD), and Pearson correlations of knowledge-sources with lay theories and likability (*N* = 177)

	*Mean (SD)*	Expression				Antecedents				Likeability	
		Grandiosity		Vulnerability		Grandiosity		Vulnerability			
		OG	CV	OV	CG	PO	PC	PO	PC	GN	VN
Popular media	2.00 (1.12)	-.04	**.17**	.06	**.22**	.05	*.13*	**.16**	.03	.08	-.06
Academic/Occupation	2.38 (1.07)	-.02	**.17**	-.01	*.13*	.04	*.13*	.*13*	-.02	.05	-.03
Personal experience	1.86 (1.19)	-.03	**.16**	-.09	**.19**	.05	**.15**	**.19**	.04	.05	-.09

*Note.* Correlations above .20, .15, and .13 are significant at*p* < .01, p < .05, and p <0.10 (two-tailed) respectively. Correlations significant at *p* < .05 and *p* < .10 are printed in bold and italic type respectively. *OG* overt grandiosity, *CV* covert vulnerability, *OV* overt vulnerability, *CG* covert grandiosity, *PO* parental overvaluation, *PC* parental coldness, *GN* grandiose narcissism, *VN* vulnerable narcissism

## Data Availability

The data to this study are available via the Open Science Framework: https://osf.io/9hwuk/.

## Footnotes

1 Note that based on the current literature, it is unclear whether *RA* should be placed within the vulnerability scales (as in the FFNI) or the grandiosity scales (as in the PNI, where the conceptually very similar *Entitlement Rage* facet has been considered to be part of grandiose narcissism in some studies and part of vulnerable narcissism in others (Pincus et al., 2009; Morf et al., 2016; Krizan & Herlache, 2017; Karakoula, Triliva, & Tsaousis, 2013).
