# Supplementary material for: Lay theories of grandiose and vulnerable narcissism
Source: Curr Psychol. Author manuscript; Available in PMC 2022 Dec 9. (PMC9715512; doi:10.1007/s12144-020-01296-w)
Supplement: ESM1 [file EMS130374-supplement-ESM1.docx]

# Appendix S1

Complementary Analyses

To test for the potentially confounding influence of sample characteristics, we controlled for sex, age, and prior diagnoses of mental disorders using path models (see figure 1 and 2). When controlling for age, the negative effect of raters’ vulnerable narcissism on likability of grandiose personality became significant (model 2: *B(without age)* = -0.11, *p(without age)* = .115, *B(with age)* = -0.17, *p(with age)* = .017). Regarding diagnoses of mental disorders, effects of lay theories about expression on likability became stronger. The finding that belief in OV (compared to covert grandiosity) led to higher likability of vulnerable personality, which was previously only significant in model 1, now also reached significance in model 2 (*W(1)* = 4.63, *p* = .032, *B_OV_* = 0.14, *B_CG_* = -0.09). All other results were stable when controlling for age, sex, and diagnoses of mental disorders.

*Figure 1.* Model 1.

*Note*. GN = grandiose narcissism, OG = overt grandiosity, CV = covert vulnerability, L(G) = likability of grandiose personality, VN = vulnerable narcissism, OV = overt vulnerability, CG = covert grandiosity, L(V) = likability of vulnerable personality.

*Figure 2.* Model 2.

*Note*. GN = grandiose narcissism, OG = overt grandiosity, CV = covert vulnerability, L(G) = likability of grandiose personality, VN = vulnerable narcissism, OV = overt vulnerability, CG = covert grandiosity, L(V) = likability of vulnerable personality.
