# Supplementary figures and images for: Lay theories of grandiose and vulnerable narcissism
Source: Curr Psychol. Author manuscript; Available in PMC 2022 Dec 9. (PMC9715512; doi:10.1007/s12144-020-01296-w)

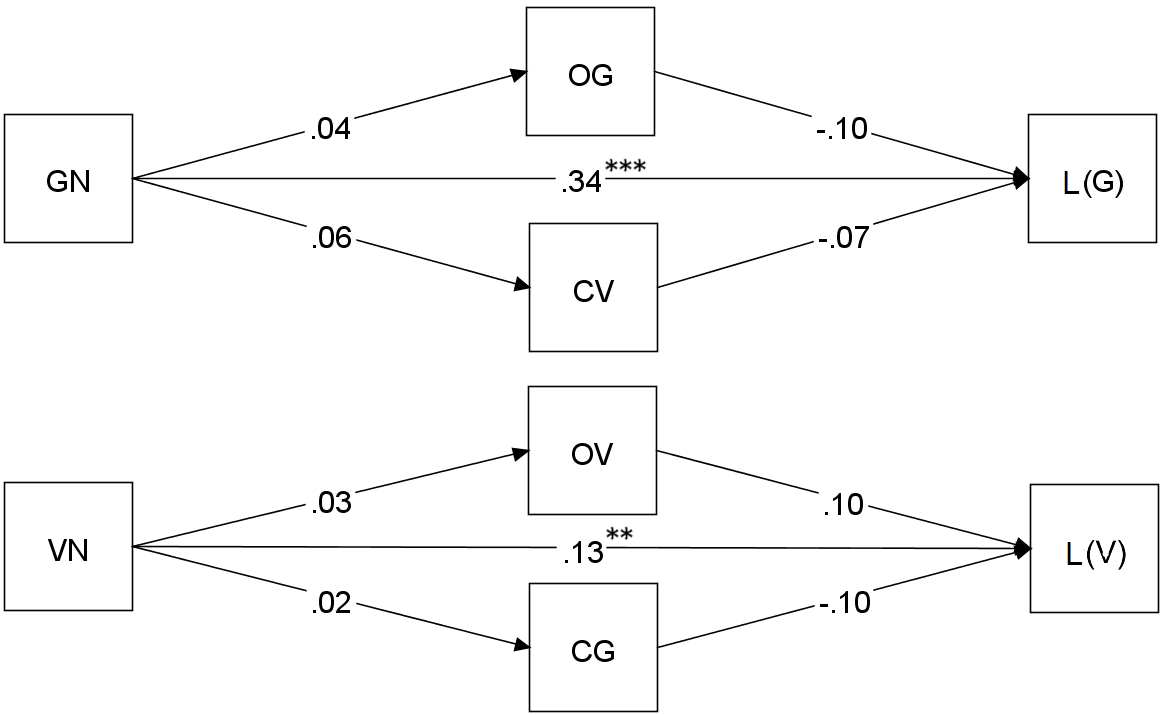

Supplement: ESM2 [file EMS130374-supplement-ESM2.png]

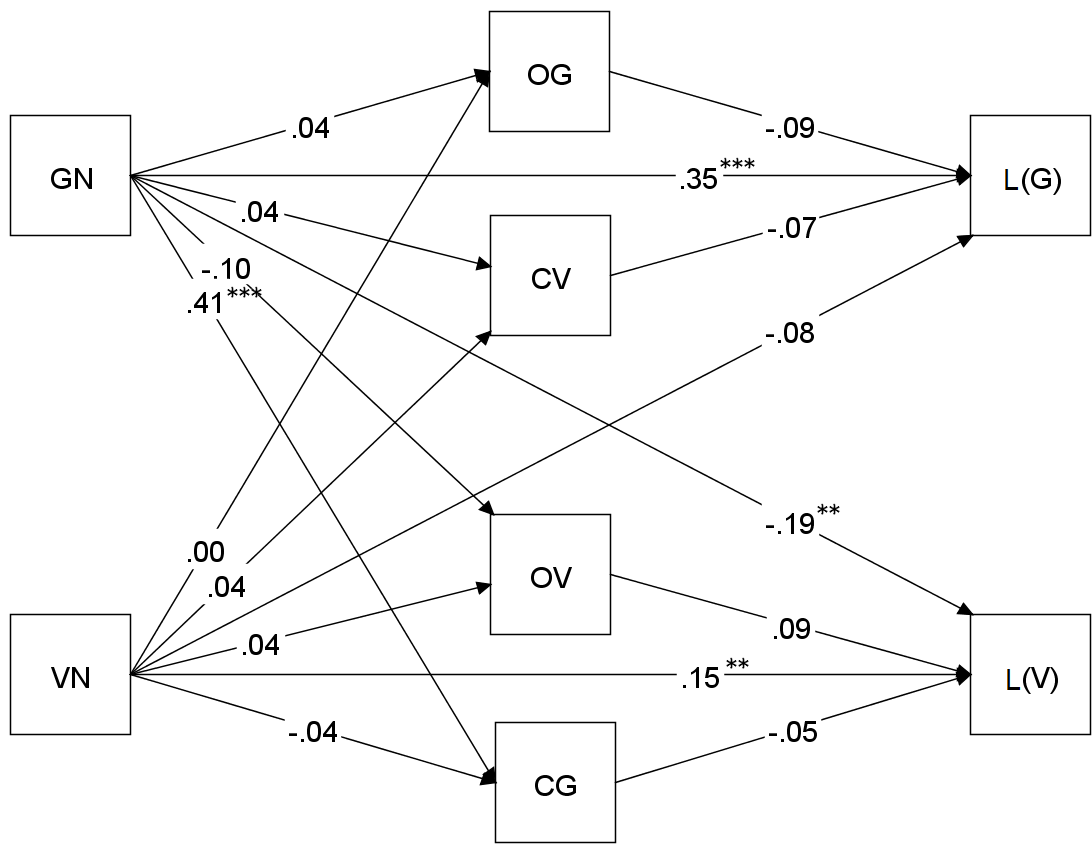

Supplement: ESM3 [file EMS130374-supplement-ESM3.png]
